# Risk factors and clinical phenotypes of Beijing genotype strains in tuberculosis patients in China

**DOI:** 10.1186/1471-2334-12-354

**Published:** 2012-12-17

**Authors:** Yu Pang, Yuanyuan Song, Hui Xia, Yang Zhou, Bing Zhao, Yanlin Zhao

**Affiliations:** 1 National Center for Tuberculosis Control and Prevention, Chinese Center for Disease Control and Prevention, Beijing, 102206, China

## Abstract

**Background:**

Beijing genotype strains are the most predominant strains in China. The aim of this study was to explore risk factors and clinical phenotypes associated with infection with Beijing genotype strains among tuberculosis patients in China.

**Methods:**

Using data and strains derived from the first Chinese national drug resistance base-line survey, we performed a statistical analysis of the relationship between different genotypes, demographic characteristics and clinical phenotypes.

**Result:**

Of patients infected with the 3634 strains for which detailed information was available, we found that people in young age groups [aged under 25 years, OR (95% CI): 1.30(1.03-1.62)], urban people [OR (95% CI): 1.18 (0.47-0.94)], or of Hui ethnicity [OR (95% CI): 1.96 (1.10-3.50)] or those needing retreatment [OR (95% CI): 1.22 (1.03-1.43)] were more likely to be infected with Beijing genotype strains compared with patients who were rural, or of Han ethnicity or those with new TB cases. In contrast, Uyghur [OR (95% CI): 0.45 (0.30-0.67)], or Zhuang ethnicities [OR (95% CI): 0.30 (0.19-0.48)], presented lower than average risk in infections with the Beijing genotype strain. In addition, a higher proportion of patients with hemoptysis [OR (95% CI): 0.81 (0.69-0.94)] and chest pain [OR (95% CI): 0.79 (0.69-0.91)] were infected with non-Beijing genotype strains than with Beijing genotype strains.

**Conclusions:**

In China, young age group, urban people, Hui ethnicity and the earlier treated patients are all high risk factors for infection with Beijing genotype strains, while Uyghur and Zhuang ethnicity are lower than average risk factors for infection. The high rate of chest symptoms occurring in non-Beijing genotype infected patients indicates that more attention should be paid to basic research on non-Beijing genotype strains.

## Background

Despite global efforts to combat tuberculosis (TB), TB is the most widespread infectious disease in the world [1]. According to a World Health Organization (WHO) report, TB causes nearly 50 to 100 million infections, 9.4 million new cases and 1.3 million deaths every year [2,3], and the situation in developing countries is especially serious. China has the second-highest TB-burden globally, behind only India [3], and data from the 5^th^ national TB epidemiological survey conducted in 2010 indicate that TB has an estimated incidence of 459 cases per 100,000 [95% confidence interval (CI): 433–484] in China, with 4.99 million prevalent TB cases (95% CI: 4.71-5.27), including 1.19 million pulmonary, bacteriologically confirmed cases (95% CI: 1.03-1.35) [4]. Although both incidence and mortality rate decreased from 2000 to 2010, TB is still a major public health problem in China due to lack of health-care facilities and other social constraints.

Among the various *M*. *tuberculosis* genotypes, the Beijing genotype is one of the most prevalent spoligotypes worldwide, especially in eastern Asia, South Africa, and northern Eurasia [5-9], and has been responsible for outbreaks of multidrug-resistant tuberculosis in several parts of the world [10-12]. As shown in many albeit not all settings, various characteristics of the Beijing genotype, including its higher virulence, ability to escape from BCG vaccine–induced protection, and significant association with multidrug resistance, may be responsible for its rapid dissemination [7,13-17]. To date, few studies have focused on the risk factors and clinical phenotypes associated with *M*. *tuberculosis* Beijing genotype strains in patients with tuberculosis. Young age (0–25 years of age) and HIV co-infection are reported to be risk factors for Beijing genotype infection in different settings [18,19]. Parwati *et al*. have shown that, in Indonesia, TB treatment is less effective against Beijing genotype strains even when drug resistance is not involved [20]. Hence, an investigation of the risk factors and clinical phenotypes associated with the Beijing genotype in regions where the Beijing genotype is the endemically prevalent strain may have important implications for Tuberculosis Control Programs. In another study [21], we spoligotyped the strains isolated from the first national drug resistance base-line survey of China [22]; 62.2% of these strains were identified as having the Beijing genotype. Here, we explore the risk factors and clinical phenotypes of Beijing genotype strains among tuberculosis patients in China, using spoligotyping data and patient information derived from the above study.

## Methods

### Patient information and bacterial strains

The protocols applied in this study were approved by the Ethics Committee of the Chinese Center for Disease Control and Prevention. All patients enrolled in the surveillance provided written informed consent, and personal information, including demographic chrematistics and clinical phenotypes.

A total of 4017 clinical strains of tuberculosis, isolated from the base-line surveillance of tuberculosis in 2007 [22], were collected from 31 provinces covered by the National TB Control Program (NTP) in China. The number of patients enrolled in surveillance was proportionally based on patient numbers in different provinces. Cultures from different survey points were transferred to the National Tuberculosis Reference Laboratory by air. Bacterial strains were then recovered on L-J medium for 4 weeks at 37°C. Genomic DNA was extracted from fresh cultures as reported previously [23]. In brief, spoligotyping was performed with an available spoligotyping kit (Isogen Bioscience BV) according to the manufacturer’s protocol [21]. Beijing genotype strains were defined as those which hybridized with all of the last nine spacer oligonucleotides (spacers 35 to 43) and none of the first 34 spacer oligonucleotides, while Beijing-like genotype strains were those that hybridized to only some of the last nine spacers.

### Data analysis

Differences in demographic and clinical characteristics between patients infected with *M*. *tuberculosis* Beijing genotype strains and patients infected with non-Beijing genotype strains were analyzed by the chi-square test and expressed as odds ratios (ORs) with 95% confidence intervals (CIs). The multivariate analysis was conducted using the surveylogistic process. All calculations were performed in SPSS 11.5 (SPSS Inc., USA), and the level of significance of univariate analysis was 0.05, and that of multivariate analysis was 0.10. The corrected R2 was used as the standard for model screening.

## Results

*M*. *tuberculosis* strains were isolated from 3634 patients who had positive sputum smear results and detailed demographic and clinical information. Spoligotyping analysis indicated that 2290 (63%) of these strains belonged to the Beijing genotype. A classification of patients infected with a Beijing genotype strain, stratified according to gender, age, ethnicity, occupation, education and income, is shown in Table 1. Overall, the percentage of men infected with a Beijing genotype strain was similar to that of women, and education level, income, family size and average family income had no influence on the prevalence of infection with Beijing genotype strains in China. We found that the prevalence of infection with Beijing genotype strains was higher among young patients [aged under 25 years OR (95% CI): 1.30(1.03-1.62)].

**Table 1:** **Demographic characteristics of tuberculosis patients according to *Mycobacterium tuberculosis *genotype**

**Characteristic**	**Beijing genotype**	**Non-Beijing genotypes**	**Odds ratios**	***P *value**
	**(2290)**	**(1344)**	**(95% CI)**	
	**N (%)**	**N (%)**		
Sex				
Male	1637(71.5%)	964(71.7%)	1.00	-
Female	653(28.5%)	380(28.3%)	1.01(0.87-1.18)	0.909
Age group (years)				
<25	403(17.6%)	202(15.0%)	1.30(1.03-1.62)	0.025
25-34	353(15.4%)	210(15.6%)	1.09(0.87-1.37)	0.452
35-44	391(17.1%)	233(17.3%)	1.09(0.87-1.36)	0.464
45-54	393(17.2%)	208(15.5%)	1.23(0.98-1.54)	0.076
55-64	316(13.8%)	209(15.6%)	0.98(0.78-1.24)	0.906
>65	434(18.9%)	282(21.0%)	1.00	-
Ethnicity				
Han	2073(90.5%)	1151(85.6%)	1.00	-
Hui	53(2.3%)	15(1.1%)	1.96(1.10-3.50)	0.021
Tibetan	12(0.5%)	5(0.4%)	1.33(0.47-3.79)	0.800
Uyghur	46(2.0%)	57(4.2%)	0.45(0.30-0.67)	<0.001
Zhuang	27(1.2%)	50(3.7%)	0.30(0.19-0.48)	<0.001
Others	79(3.4%)	66(4.9%)	0.67(0.48-0.93)	0.017
Occupation				
Rural	1470(64.2%)	912(67.9%)	1.00	-
Urban	820(35.8%)	432(32.1%)	1.18(1.02-1.36)	0.025
Education Level				
Illiterate	465(20.3%)	274(20.4%)	1.00	-
Primary school	686(30.0%)	448(33.3%)	0.90(0.75-1.09)	0.308
Middle school	719(31.4%)	432(32.1%)	0.98(0.81-1.19)	0.846
High school	319(13.9%)	148(11.0%)	1.27(0.99-1.62)	0.063
University	101(4.4%)	41(3.1%)	1.45(0.98-2.15)	0.069
Income (USD)				
<348	446(19.5%)	244(18.2%)	1.00	-
348-583	516(22.5%)	293(21.8%)	0.96(0.78-1.19)	0.746
584-2778	1112(48.6%)	685(51.0%)	0.89(0.74-1.07)	0.211
2779-6944	181(7.9%)	109(8.1%)	0.91(0.68-1.21)	0.513
>6944	35(1.5%)	13(1.0%)	1.47(0.77-2.84)	0.275
Number of family members (Person)				
1-2	555(24.2%)	300(22.3%)	1.00	-
3-5	1401(61.2%)	850(63.2%)	0.89(0.76-1.05)	0.170
>5	334(14.6%)	194(14.4%)	0.93(0.74-1.17)	0.564
Average family income (USD)				
<116	495(21.6%)	272(20.2%)	1.00	-
116-336	758(33.1%)	436(32.4%)	0.96(0.79-1.16)	0.665
337-560	321(14.0%)	185(13.8%)	0.95(0.76-1.20)	0.720
>560	716(31.3%)	451(33.6%)	0.87(0.72-1.05)	0.164

CI, confidence interval.

Rural, the people who live in rural communities.

Statistical analysis revealed that the percentage of people infected with Beijing genotype strains differed according to ethnicity. Compared with the percentage of Beijing genotype strains isolated from people of Han ethnicity, the percentage of strains isolated from people of Hui ethnicity [OR (95% CI): 1.96 (1.10-3.50)] was higher, while the percentage of strains isolated from people of Uyghur [OR (95% CI): 0.45 (0.30-0.67)], Zhuang [OR (95% CI): 0.30 (0.19-0.48)] and other Chinese ethnicities [OR (95% CI): 0.67(0.48-0.93)] was significantly lower. There were no statistically significant differences in the rate of infection with Beijing genotype strains between Han and Tibetan ethnicities.

Occupations associated with infection with Beijing or non-Beijing strains were compared. As shown in Table 1, there were significantly more urban people [OR (95% CI): 1.18 (0.47-0.94)] among tuberculosis patients infected with Beijing genotype strains, indicating that urban people are a risk factor for infection with strains of the Beijing genotype. We also performed the multivariate analysis towards the distribution of people infected with Beijing genotype strains based on the univariate analysis. As shown in Table 2, the analysis data revealed that young age group, urban people and Hui ethnicity are all high risk factors for infection with Beijing genotype strains, while Uyghur and Zhuang ethnicity are lower than average risk factors for infection, similar to univariate analysis.

**Table 2:** **Multivariate analysis of demographic characteristics of tuberculosis patients according to *Mycobacterium tuberculosis *genotype**

**Characteristic**	**Adjusted ORs (95% CI)**	***P *value**
Age group <25 years	1.35(1.03-1.78)	0.031
Hui Ethnicity	1.90 (1.06-3.41)	0.032
Uyghur Ethnicity	0.48(0.32-0.72)	<0.001
Zhuang Ethnicity	0.34(0.21-0.54)	<0.001
Urban People	1.08(0.91-1.28)	0.389

OR, odds ratio; CI, confidence interval.

Clinical phenotypes of patients infected with Beijing genotype or non-Beijing genotype strains are shown in Table 3. The percentage of cases requiring retreatment [OR (95% CI): 1.22 (1.03-1.43)] was higher among people infected with Beijing genotype strains than among people infected with non-Beijing genotype strains. We also examined relationship between infection with Beijing genotype strains and clinical symptoms. A cough was the most common symptom for both Beijing and non-Beijing genotype strain infected patients, and there was no significant difference in the incidence of this symptom between these two groups. A significantly lower percentage of Beijing genotype infected patients with hemoptysis [OR (95% CI): 0.81 (0.69-0.94)] and chest pain [OR (95% CI): 0.79 (0.69-0.91)] was observed, using patients infected with non-Beijing genotype strains as a reference. In contrast, fever [OR (95% CI): 1.25 (1.09-1.43)] was more frequently observed in patients infected with Beijing genotype strains than in those infected with non-Beijing genotype strains. In addition, TB contact history and the use of DOTS monitoring did not result in significant differences between the two groups.

**Table 3:** **Clinical characteristics of tuberculosis patients according to *Mycobacterium tuberculosis *genotype**

**Characteristic**	**Beijing genotype**	**Other genotypes**	**Odds ratios**	***P *value**
	**(2290)**	**(1344)**	**(95% CI)**	
	**N (%)**	**N (%)**		
Medical history				
New case	1748(76.3%)	1071(79.7%)	1.00	-
Retreatment case	542(23.7%)	273(20.3%)	1.22(1.03-1.43)	0.021
History of tuberculosis contact				
Yes	1864(81.4%)	1081(80.4%)	1.00	-
No	426(18.6%)	263(19.6%)	0.94(0.79-1.11)	0.483
Live in area with DOTS implemented				
Before 2000	1276(55.7%)	793(59.0%)	1.00	-
2000 or after	1014(44.3%)	551(41.0%)	1.14(1.00-1.31)	0.056
Cough				
No	122(5.3%)	66(4.9%)	1.00	-
<3 weeks	382(16.7%)	214(15.9%)	0.97(0.69-1.36)	0.862
≥3 weeks	1786(78.0%)	1064(79.2%)	0.91(0.67-1.24)	0.586
Hemoptysis				
No	1715(74.9%)	949(70.6%)	1.00	-
Yes	575(25.1%)	395(29.4%)	0.81(0.69-0.94)	0.005
Fever				
No	1167(51.0%)	758(56.4%)	1.00	-
Yes	1123(49.0%)	586(43.6%)	1.25(1.09-1.43)	0.002
Chest pain				
No	1549(67.6%)	837(62.3%)	1.00	-
Yes	741(32.4%)	507(37.7%)	0.79(0.69-0.91)	0.001

OR, odds ratio; CI, confidence interval.

## Discussion

To the best of our knowledge, this is the first report of risk factors and clinical phenotypes associated with the Beijing genotype in China. Many of the investigations performed in other countries are limited by sample size or loss of detailed epidemiological information [12,13,24,25]. Using data for 3634 strains identified in the first national drug-resistance surveillance of China, our data describe the detailed population structure of both Beijing and non-Beijing genotype strains.

According to our study, the Beijing genotype is the most predominant genotype in China, and several factors have an influence on its distribution among the population. Of all the factors, the correlation between Beijing genotype with MDR and fluoroquinolone resistance might influence the overall risk factors for Beijing spoligotypes compared to other genotypes [21]. In addition, a previous study in Vietnam indicated that the Beijing genotype is associated with young patients (aged less than 25 years), while research performed in other countries has suggested that infection with the Beijing genotype showed no association with a patient’s age [13]. Consistent with results from Vietnam, we found that young age (less than 25 years) showed a strong association with infection by the Beijing genotype, suggesting that the Beijing genotype will tend to become dominant in the population in China, possibly because young people tend to engage in greater levels of social interaction. van Soolingen *et al*. hypothesized that BCG vaccination may be one of the selective forces implicated in the successful spread of the Beijing genotype [26]. This hypothesis is supported by results from other studies [27]. There is speculation that the Beijing genotype strains may have diverged from a common ancestor in the recent past. Although the exact date of their divergence cannot be calculated, it is thought that this evolutionary process has been completed in less than a century [26]. Hence, one possible explanation for the low spread of the Beijing genotype in the older population is that older patients previously infected with the non-Beijing genotype are resistant to the recently-emerged Beijing genotype strain. Additionally, another possible explanation could be an increased ongoing transmission in the society of this form of TB, which would be reflected in a higher prevalence in the young. Further study is required to confirm the hypothesis.

In addition to age, infection with the Beijing genotype was associated with ethnicity. Compared with Han, people of both Uyghur and Zhuang ethnicities showed a lower than average risk of infection with the Beijing genotype. In a previous study we showed that the percentage of Uyghur patients infected with CAS1-DEHLI genotype strains was significantly higher than that of other ethnicities [21], and suggested that this may be due to the frequent exchange between Uyghur people and ethnicities from several neighboring Central Asian countries where the CAS1-DEHLI genotype is highly prevalent, since they share similar cultural traditions. Since the Zhuang ethnicity is mainly distributed in Guangxi Province in South China, the relatively low incidence of the Beijing genotype is consistent with the relatively low percentage of Beijing genotype strains in Southern China [21]. In contrast to other ethnic minorities, Hui ethnicity is defined rather based on cultural or religious reasons instead of genetic one. Hence, the special traditions and ways of living in Hui ethnicity may be responsible for the high percentage of Beijing genotype. However, a larger study is needed to answer additional questions about the distribution of Beijing genotype and non-Beijing genotype strains among different ethnicities.

With respect to occupation, we found strong associations between urban people and infection with Beijing genotype strains. In China, rural people are usually living in rural area with less density of population, who depend on argricultural activities for their livelihoods, and therefore their mainly work condition is outside the building; whereas urban people tend to live in urban areas with more density of population and always work inside the crowd public environment, which contribute to Beijing infection of urban people. In addition, many studies have suggested that the Beijing genotype is associated with resistance to anti-tuberculosis drugs [18,28-30]. Due to the unbalanced distribution of health care resources in China [31], misuse and overuse of antibiotics is more common in urban areas than in rural areas in China, and this may account for the higher prevalence of drug-resistant Beijing genotype strains among urban people.

In addition to demographic characteristics, patients infected with Beijing genotype strains presented different clinical profiles compared with those infected with non-Beijing genotype strains. Both hemoptysis and chest pain are common manifestations of pulmonary tuberculosis [32]; moreover, massive hemoptysis is a life-threatening condition with a poor treatment outcome in active tuberculosis patients [33]. In this study, we observed that 25.1% and 32.4% of patients infected with Beijing strains had hemoptysis and chest pain, respectively, compared with 29.4% and 37.7%, respectively, of those infected with non-Beijing strains. These differences were statistically significant. That both symptoms have a lower occurrence in patients infected with the Beijing strain is reasonable as the two symptoms often occur together in the same patient. Our data indicate that chest symptoms occur more frequently in patients infected with non-Beijing strains. Our results are in agreement with a study performed in Singapore which demonstrated that there is a significantly higher frequency of cavitary disease in patients infected with non-Beijing genotype strains [25]. Nevertheless, in studies on the CXR presentation of patients completed in Indonesia and in the Netherlands [12,20], the authors did not find any significant difference between the two groups. While more attention has been given to the Beijing genotype than to other genotypes due to the association of its supposed higher virulence with relapse and treatment failure in humans [13,20,34-36], our findings suggest that patients infected with non-Beijing genotype strains may have more serious chest phenotypes.

Fever is a typical clinical symptom of tuberculosis patients, and recent findings have revealed that the frequency with which fever is observed is more frequently reported in non-Beijing genotype infected patients than in patients infected with the Beijing genotype [25]. In contrast, we found that fevers occurred in a significantly higher proportion of patients infected with Beijing genotype strains in China than in those infected with non-Beijing genotype strains. Our results are in agreement with a cohort study by Parwati *et al*. which showed that more patients infected with Beijing genotype strains developed fever during treatment [20].

## Conclusion

In summary, we have shown that ethnicity, occupation and earlier treated patients are risk factors for infection with Beijing genotype strains, and identified clinical phenotypes that are strongly associated with the genotype of the strain with which a patient is infected. Our data suggest that urban people, Hui ethnicity and earlier treated patients are all high risk factors, while older age group, and Uyghur or Zhuang ethnicity show a lower than average risk for infection with the Beijing genotype. Other characteristics, including sex, economic situation, contact history and implementation of DOTS monitoring did not vary between patients infected with Beijing and non-Beijing genotype strains. In addition, the high proportion of hemoptysis and chest pain appearing in non-Beijing genotype infected patients indicates that more attention should be given to basic research on non-Beijing genotype strains.

## Competing interests

The authors declare that they have no competing interests.

## Authors' contributions

Conceived and designed the experiments: YLZ YP HX YYS. Performed the experiments: YP YYS HX YZ BZ. Analyzed the data: YP HX. Wrote the paper: YLZ YP. All authors read and approved the final manuscript.

## Pre-publication history

The pre-publication history for this paper can be accessed here:

http://www.biomedcentral.com/1471-2334/12/354/prepub
